# Supplementary material for: Rhizospheric soil chemical properties and microbial response to a gradient of Chromolaena odorata(L) invasion in the Mount Cameroon Region
Source: PLoS One. 2024 Nov 27;19(11):e0312199. doi: 10.1371/journal.pone.0312199 (PMC11602113; doi:10.1371/journal.pone.0312199)
Supplement: Attachment — Submitted filename: Rhizospheric_Reviewed.doc [file pone.0312199.s002.doc]

**Rhizospheric Soil Physicochemical Properties and Microbial Response to a Gradient of *Chromolaena odorata*(L) Invasion in the Mount Cameroon Region**

Victor Nzengong Juru^13¶^, Lawrence Monah Ndam^*234&^, Blaise Nansingnyuy Tatah^2¶^, Beatrice Ambo Fonge^14&^

1 Department of Plant Science, University of Buea, Cameroon

2 Department of Agronomic and Applied Molecular Science, University of Buea, Cameroon

3 Institute for Nature, Health and Agricultural Research (INHAR), Buea, Cameroon

4 Agroecology Research Group, University of Buea, Cameroon.

*Corresponding author: [lawrencendam3@gmail.com](mailto:lawrencendam3@gmail.com)

**Abstract**

This study investigates the impacts of *Chromolaena odorata* invasion gradients on rhizospheric soil physicochemical properties and microbial response in the Mount Cameroon Region. Allelopathic potentials of this weed have had little attention, leading to changes in soil properties and microbial communities. Forty-eight soil samples at four different degrees of invasion based on species coverage within subplots in four study areas were collected and rhizospheric soil physicochemical properties, microbial load, phosphatases enzyme activities and secondary metabolites were evaluated. At medium-degree invasion, rhizospheric soil concentrations of P, K, and Fe increased with arbuscular mycorrhizal fungi (AMF) colonization and phosphatases enzyme activities. Soil C, N, and organic matter were significantly increased at high-degree invasion, supporting the use of the plant as a fallow crop. Acid phosphatase activity ranged from 0.69 to 0.90 mmol h^-1^ kg^-1^ and was significantly different at different degrees of invasion. AMF colonization ranged from 23.33 to 50.00%, with a strong positive correlation between AMF colonizationand phosphatase activity. Soil bacterial load was high (460000CFU/g - 670000CFU/g), mostly *Staphylococcus* with health concerns about its spread. The invasion situation had no significant effects on soil bacterial load, but high-degree invasion significantly increased fungal load. Low-degree invaded soils had high saponin (24.55±0.00 mg/g), flavonoid (47.75±0.00 mg/g) and tannin (28.68±0.07 mg/g) concentrations. This degree of invasion is likely the most potential for bioremediation of heavy metal-polluted soils and collection of parts for medicinal purposes. However, the study recommends detailed studies on how gradients of invasion impact rhizospheric soil properties under controlled environments, molecular identification of microbial communities, and quantification of secondary metabolites in plant parts at different invasion gradients.

**Keywords:** *Chromolaena odorata*, Invasion, Rhizospheric soil, Microorganisms, phosphatase, secondary metabolites

1. **Introduction**

The globalisation of human activities has caused plants to spread beyond their natural habitats, resulting in a significant change in ecosystem function and structure, and a change in soil chemical composition [1-2]. This biological invasion is the second-largest threat to biodiversity after habitat destruction and is one of the top environmental concerns [3]. The increase in invasive species and their irreversible impact on ecosystems is a major concern for conservationists worldwide [4]. The slow and subtle changes caused by invasive species may be difficult to detect until they reach their consolidated state and exert maximum negative effects [5].

Invasive species release root exudates that stimulate soil microbial communities and increase nutrient cycling [6]. Invasive plants alter soil microbial community structure, which impacts native plant performance [7-8] by increasing pathogenic microbes and decreasing beneficial ones required for the fitness of native plants [9]. Invasive plants invade environments by altering the soil microbial community in their rhizosphere. For instance, low and high degrees of *Wedelia trilobata* invasion increase the richness of soil fungal communities but do not affect soil bacterial communities [10]. Invasive plants can form arbuscular mycorrhizal fungi (AMF) associations or degrade local mycorrhizal fungi. This often favours the proliferation of generalist AMFs to local specialist ones [11]. Mycorrhizal fungi are crucial in optimising soil aggregation and are essential in water and nutrient uptake, including phosphorus, zinc and copper. In phosphorus-deficient tropical environments, these fungi participate in phosphorus fixation, comprising phosphorus recovery in the soil profile [12].

The rate of phosphorus mineralisation is governed by microbial activity and plant-synthesised enzymes [13]. However, some invasive plant species can boost the activity of phosphatase enzymes in rhizosphere soil due to the higher phosphorus uptake [14]. Phosphatases are a class of enzymes that break ester-phosphate bonds, resulting in the release of phosphate (P) that can be taken up by plants or microorganisms [15]. Moreover, the activities of phosphatases, including acid and alkaline phosphomonoesterase, are influenced by soil properties, soil organism interactions, plant cover, and leachate inputs [13]. *Pseudomonas sp.* produces phosphatase that stimulates enzyme synthesis by intact roots, releases inorganic phosphate from organic and complex inorganic materials, and plays an essential role in the phosphorus cycle [16]. It also solubilises organic and inorganic phosphates, thereby supporting plant growth [17].

*Chromolaena odorata* (L) R. King and H Robinson (Asteraceae) is a perennial shrub that originated from Central and South America [18]. However, it has become an invasive weed in tropical and subtropical regions of Africa and other parts of the world, spreading rapidly in open areas such as roadsides, riverbanks, disturbed forests, and plantations [19]. This weed exhibits a high reproductive capacity and has several common names in different regions, such as Siam weed, devil weed, French weed, communist weed, hagonoy, co hoy, triffid weed, Bitter bush, Christmas Bush, Camphur Grass, Common Floss Flower, and achacasala in Cameroon. [20-21] provide an overview of the common names associated with this weed.

*Chromolaena odorata*, is one of the world's worst invasive weeds that pose a severe threat to agriculture, biodiversity, and the environment. The plant releases allelochemicals such as flavonoids, terpenoids, and alkaloids that alter nutrient fluxes and even modify soil microbial communities [22]. The weed outcompetes native plants via light competition and accumulation of soil-borne native pathogens and has a fast growth rate [23]. Although *C. odorata* has been used as a medicinal weed and fallow crop in the Mount Cameroon Region, studies have established that its eradication, containment, exclusion, or population reduction is difficult due to the limited management interventions that tackle the general ecosystem [24]. Its proliferation in forest gaps, along riversides, roadsides, and plantations results in the rapid recolonizationof agricultural fields, causing significant economic losses [21]

Proper management of the species will depend on a precise evaluation of all stages of the invasion process in natural ecosystems [25]. Furthermore, more studies are necessary to evaluate the plant's impact on below-ground biodiversity and its ecological ramifications on restoration activities in invaded ecosystems. Although previous studies have focused on the plant's effect on soil chemical and physical properties, more attention needs to be given to the microbial and physical properties that interact within the rhizosphere to establish the plant's complete impact [26]. Finally, there is a need for more data on the allelopathic potential of *C. odorata* at different degrees of invasion and their effects on soil microbial communities, even on phosphatase activities during invasion, as noted by [10]. Understanding the effects of *C. odorata* invasion on soil properties and microbial communities is critical in the development of better and more effective management strategies aimed at mitigating the impacts of invasive species and maintaining the ecological health of the Mount Cameroon region. Hence, the study aimed to assess the impact of *C. odorata* invasion on soil physicochemical properties, microbial load, and rhizospheric biological activities in the Mount Cameroon Region. The study had four specific objectives: (i) to assess the effects of different degrees of *C. odorata* invasion on soil physicochemical properties, (ii) to evaluate the effects of *C. odorata* invasion on soil microbial load, (iii) to investigate the effects of *C. odorata* invasion on phosphatases activities, and (iv) to analyse the secondary metabolites released to rhizospheric soils by *C. odorata* at different degrees of invasion.

**2. Materials and Methods**

**2.1 Site Description**

The Mount Cameroon Region (MCR) is found in the southwest region of Cameroon and covers a surface area of 5695.5 km^2^, stretching from latitude 3^o^57^'^ to 4^o^28^'^ N and longitude 8^0^58'to 92^o^4^'^ E [27]. The region stretches just about 20 m above sea level to a height of 4100 m, called Mount Cameroon, situated at 4^o^13^'^ N, and 9^0^10^'^ E. This is the highest point in West and Central Africa. The climate is humid with annual rainfall ranging from 2085 mm to 10000 mm. The mean temperature is 25^o^C, which decreases by 0.6^o^C per 100 m ascent [28]. According to the Institute of National Statistics of Cameroon (2010), the population of the South West region is 1384300 people, with about 400,000 people living around the Mount Cameroon National Park area [29]. The region hosts several plantations owned by the Cameroon Development Cooperation (CDC) and small scheme holders.

This study was conducted in Buea, Bakingili, Limbe and Idenau, localities around the Mount Cameroon National Park (Fig. 1). Field surveys were done in the two major seasons between September 2019 and November 2020 from three land uses; roadsides, farmlands and forests. Accessibility, tourism, anthropogenic activities, historical facts, and safety were paramount in site selection.

**Fig. 1:** Maps of Cameroon and the Mount Cameroon Region showing study sites

**2.2. Experimental Design**

The modified Whittaker method was used to lay 120 permanent plots of size 20 x 50 m, with 10 plots per land use (roadside, farmland and forest) and 30 per site. Along the inside border, subplots of size 2 x 5 m were placed systematically in alternate corners and a 5 x 20 m subplot was placed at the centre. All plots with *C. odorata* were tagged and noted. In April 2022, rhizospheric soil samples were collected at different degrees of invasion from the four sample areas using slightly modified methods [30]. Each sample area was divided into four sites based on the degree of *C. odorata* invasion, using coverage of the species within each subplot as (i) uninvaded (0%, ND), (ii) low degree (<35%, LD), (iii) medium degree (50%, MD) and (iii) high degree (>75%, HD) (Fig. 2). Forty-eight soil samples within 5 cm of *C. odorata* rhizosphere from the four study areas at the four different degrees of invasion were collected in sealed bags and stored at about 10ºC. The soil samples were collected from Buea (N 04º7´52.97“/E 09º13´24.27“to N04º12´27.42“/E 09º16´20.31“) and altitude 781–971 m a.s.l), Limbe (N 03º35´59.59“/E09º15´14.28“to N04º2´10.25“/E09º5´53.25“) and altitude 63-181 m a.s.l), Bakingili (N 04º4´11.89“/E 09º2´11.93“ to N 04º3´3.56.99“/E 09º1´50.77“) and altitude 9-24 m .a.s.l and Idenau (N04º14´21.82“/E08º59´45.91“ to N 04º12´13.32“/E08º59´53.95“) and altitude 9-52 m a.s.l. The samples were homogenised based on the degree of invasion and study area (4 study areas x 4 degrees of invasion), resulting in sixteen treatments, which were taken to the laboratory for analysis after sieving with a 2 mm sieve.

(a) Uninvaded (0%)	(b) Low Degree invasion (<35%)	(c) Medium degree invasion (50%)	(d) High degree invasion (>75%)

**Fig. 2:** Different degrees of invasion of C odorata based on cover percentage per plot

**2.3. Soil Physicochemical Properties**

Standard methods were used to analyse soil physicochemical properties, including pH, moisture %, organic matter, organic C, N, Mn, P, Fe, K, Ca, and Mg. The pH was measured using the Thermo-Russell pH meter following the method of Black [31], where 10g of 2mm soil samples were weighed into beakers, 25 mL of KCl and distilled water were added, and a soil solution was made. The solution was stirred and left overnight, and the pH was measured the following day by dipping the calibrated electrode into the sample. Total nitrogen was determined by colourimetry from absorbance ƛ650nm using a spectrophotometer. Soil samples were digested at 370°C with concentrated H_2_SO_4_ in the presence of sodium sulphate/selenium catalyst (Kjeldal method), and absorbance was used to determine total nitrogen against a standard solution [32]. Organic carbon was determined by the Walkley-Black method, where the organic carbon in the soil was oxidised with K_2_Cr_2_O_3_ and concentrated H_2_SO_4_, and the colourimetric readings were compared with the blank (sucrose and water) [31]. Available phosphorus in the soil was determined colourimetrically using the ammonium molybdate blue colouration method. Mg and other bases (Ca, K, and Na) were extracted using ammonium acetate and determined colourimetrically on a spectrophotometer using the "Titan yellow method". The concentrations of Mn, Fe, Na^+^, K^+^, and Ca^2+^ were determined using the Gallenkamp flame photometer by way of Atomic Emission Spectrometers, involving the thermal excitation of free atoms.

**2.4 Microbial load**

**2.4.1 Bacterial count**

To obtain the bacterial count of soil samples, one gram of soil sample was mixed with 10mL of distilled water and serially diluted nine times (10^-1^ to 10^-9^) by adding sterile distilled water to each test tube, homogenising the preparations at each step. From the preparations, 1 mL of soil sample was mixed with 9 mL of sterile distilled water to make 9-fold serial dilutions of each preparation, and the serial dilutions were made up to 10^9^ dilutions. A volume of 0.1 mL from the seventh (10^7^), eighth (10^8^), and ninth (10^9^) dilutions were each inoculated in duplicate on a nutrient agar plate using the spread plate technique. The inoculated plates were incubated at a temperature of 37˚C for 24 hours. The same serial dilutions were made for other samples and they were also incubated at the same temperature and time. After incubation, the bacterial colonies formed on the nutrient agar media were counted and recorded, and the plates were labelled with the dilution number. The total number of bacteria per mL of the original sample was calculated by multiplying the number of colonies counted by the dilution factor (Eqn. 1) [33]. To calculate the colony-forming units (CFU) of bacteria per gram of soil, the number of colonies was multiplied by the reciprocal of the dilution factor (Eqn. 2).

Total number of bacteria per mL = number of colonies counted × dilution factor………………..(1)

CFU/g = colonies number × 10000 ………………………………………………………………..(2)

**Bacteria culture, phenotypic and biochemical characteristics**

The working space was cleaned and disinfected with 75% alcohol. Using a balance, 1g of soil sample was weighed and added to a test tube containing 10 mL of sterile 0.9% NaCl. The test tube was shaken vigorously to separate the bacteria from the soil particles. Using a micropipette and a sterile tip, 1000µL of re-suspended soil was transferred to the tube labelled 10^0^ dilutions. It was vortexed for 30 seconds to thoroughly mix the cells into the saline. This was followed by 10^-1^, 10^-2^ and 10^-3^ serial dilutions. This procedure was done for all 4 samples. Nutrient agar was prepared by measuring 14.0 grams into 500ml of water and autoclaved. The soil sample dilutions were poured into sterile petri dishes and labelled. The agar was removed from the autoclaved and allowed to cool, then poured into the petri dishes containing the samples and allowed to solidify. The petri dishes were incubated at 37°C for 24 hours. After 24 hours the petri dishes were removed from the incubator, and the phenotypic characteristics were recorded. Catalase test was done by scraping a bacteria colony from the agar on a slide, 1 drop of hydrogen peroxide was added and results were recorded. Gram staining was carried out using a clean, grease-free slide. A smear of suspension was placed on the clean slide with a loopful of samples. The slide was air-dried and heat-fixed. Crystal violet was poured and kept for about 30 seconds to 1 minute and rinsed with water. It was flooded with the gram’s iodine for 1 minute, washed with 95% alcohol for about 10-20 seconds and rinsed with water. Safranin was added for about 1 minute and washed with water. The preparation was air-dried blotted dry and Observed under the microscope.

**2.4.2. Fungal count and culturing**

The working space was cleaned and disinfected with 75% alcohol. PDA (potato dextrose agar) was prepared by weighing 5g of agar into 100 mL of water in a conical flask. The prepared agar was autoclaved and serial dilutions were made for the 4 soil samples. The dilutions 10^-3^ to 10^-5^ were used. To calculate the colony-forming units (CFU) of fungus per gram of sample, the number of colonies was multiplied by the reciprocal of the dilution . The CFU/g was calculated as in Eqn. 2

1mL of the sample was poured into sterile petri dishes and labelled. The agar was removed from the autoclave and allowed to cool. After cooling the agar was poured into the petri dish containing the sample and left to solidify. The culture was kept in a cool, moist area for two weeks for growth to occur [34]. After two weeks the phenotypic characteristics were recorded and microscopy was done by putting a sample of fungi on the slide and stained with methylene blue then viewed under the microscope.

**2.4.2.1. Determination of arbuscular mycorrhizal colonisation**

Triplicate samples of fine roots of *C. odorata* were collected from each degree of invasion and each site, placed in sealed bags and promptly transported to the laboratory. Assessment of arbuscular mycorrhizal fungi (AMF) root colonisation [[35]. Segments of root samples measuring 1-2 cm were placed in a 5% KOH solution for 24 hours at room temperature, rinsed three times with water on a fine sieve, acidified in 10% HCl (v/v) for 15 minutes, and stained with 0.01% (w/v) fuchsine acid for 24 hours at room temperature. Random root segments of stained samples were selected, and three replicates of 10 roots per slide were assessed for the presence of AMF structures (vesicles, arbuscules, and hyphae) using an optical microscope (Biological compound microscope with replaceable LED light, OMAX 40X-2500X Trinocular, Germany). The mycorrhizal frequency (F %) was obtained as the ratio of colonised root fragments to the total number of observed root fragments.](https://yousearch.canny.io/)

[**2.5. Determination of phosphatase activities**](https://yousearch.canny.io/)

The method for quantifying rhizosphere acid phosphatase activity followed [36]. Five plants were randomly selected at each invasion degree for acid phosphatase activity assessment. A spade was used to dig approximately 5cm around each plant at a depth of 20cm. One gram of root-adhering soil was collected from each plant and pooled to create a composite sample of 5g. A microcentrifuge tube was filled with 1g of the composite sample, to which 0.5 mL of 100 mM phosphate buffer was added, followed by 10 mM of P-nitrophenyl phosphate (p-NPP) in 100 µL solution. The final reaction mixture volume was adjusted to 1 mL with distilled water, and the tube was vortexed for 2 minutes at room temperature and incubated at 37°C for 1h with shaking (100rpm). After incubation, the samples were centrifuged at 10,000 rpm for 5 min, and the clear supernatants were transferred to clean test tubes and treated with 2 mL of 1 M NaOH. The resulting yellow-coloured filtrate was analysed using a colourimeter (Klett colourimeter, Clinical model, 800-3, 115 VAC) at λ = 430nm. The concentration of soluble protein in the supernatant was measured using the Bradford reactive procedure [37] and the specific activity of the soil acid phosphatase was estimated in mg h^-1^ g^-1^ using the formula:

 …………………………………………………………………(3)

Where 10 = constant, C = concentration of soluble protein and 17800 is the molecular extension coefficient of phosphatase

**2.6. Secondary metabolites in rhizospheric soil (Quantitative Allelochemicals)**

**2.6.1. Determination of total phenolic content (TPC)**

The Folin-Ciocalteu colourimetric method [38] was employed to determine the total phenolic content of the aqueous soil samples. Specifically, 0.2 mL of soil sample was mixed in a test tube with 1.5 mL of Folin-Ciocalteu reagent and incubated for 5 minutes at room temperature. After that, 1.5 mL of a 6% sodium carbonate solution was added to the mixture, which was re-incubated for 90 minutes at room temperature. A quartz cuvette at 725 nm measured the absorbance of the resulting blue colour. Meanwhile, gallic acid standards were prepared by dissolving 0.75 mg of gallic acid in 5 mL of distilled water. Different volumes (0, 0.1, 0.2, and 0.3) of the gallic acid standard were pipetted into four labelled test tubes, and distilled water was added to each to make up the volume to 0.3 mL. A 2.25 mL of Folin-Ciocalteu reagent was added to each test tube, followed by incubation for 5 minutes at room temperature. The mixture was re-incubated for 90 minutes at room temperature after adding 2.25 mL of 6% sodium carbonate solution. A quartz cuvette was used to read the absorbance of the resulting blue colour at 725 nm. This was used to draw a standard curve from which the total phenolic content of the samples was estimated. The total phenolic content of the samples was expressed as µgGAE/g soil sample (Fig. 3).

**Fig. 3:** Calibration curve of gallic acid in the determination of Phenolic content of samples

**2.6.2. Total Flavonoid Content**

Specifically, 0.15g of soil sample was homogenised with 10 mL of an extraction solvent containing methanol, distilled water, and acetic acid (in a 140:50:10 v/v ratio). The mixture was filtered using Whatman No. 1 filter paper. Next, 0.2 mL of the sample solution was mixed with 1.8 mL of distilled water and 1 mL of an aluminium chloride reagent consisting of 133 mg of aluminium chloride crystals and 400mg of sodium acetate dissolved in 100 mL of the extraction solvent). The resulting mixture was vortexed, and the absorbance was read at 430 nm against a blank. The amount of flavonoids was calculated using a standard solution of rutin (0.1mg/mL), and the results were expressed in milligrams of Quercetin per gram of soil sample (Fig. 4).

**Fig. 4:** Calibration curve of rutin in the determination of flavonoid content of samples

**2.6.3. Tannin Content**

To determine the tannin content in the soil samples, the spectrophotometric method involving acidified vanillin was employed. One gram of each soil sample was mixed with 15 mL of acetone (10% acetic acid) and stirred for 15 minutes before filtering and diluting 20 times with distilled water. Next, 1 mL of the diluted mixture was added to each test tube coated with aluminium foil to exclude light. Then, 3 mL of a freshly prepared solution of 4% vanillin in ethanol (w/v) was added, and the mixture was stirred. Following this, 1mL of concentrated HCl was added to each tube, and the solution was kept at room temperature for 15 minutes before reading the absorbance at 500nm against a blank. The amount of tannins was determined using a standard solution of tannic acid (5mg/mL), and the results were expressed in milligrams of tannic acid per gram of sample (Fig. 5).

**Fig. 5:** Calibration curve of tannic acid for determination of total tannins

**2.6.4. Saponin content**

The method used in this assay was based on the oxidation of triterpene saponins using vanillin, with strong sulphuric acid as the oxidising agent to break apart the aglycone from the complex saponin molecule. To estimate the total saponin content in the soil samples, we followed the method described by [39], which involves reacting the saponins with a sulphuric acid-vanillin reagent. Specifically, 0.5 mL of the sample was added to a Falcon tube containing 0.5 mL of vanillin (8% w/v) and 4.0mL of sulphuric acid (72% w/v). The mixture was incubated at 60°C for 15 minutes in a water bath and later cooled in an ice bath for 10 minutes. The absorbance of the sample was read at 560nm, using aescin as a reference standard. The total saponin content (mg/g soil sample) was estimated using the calibration curve for aescin (Fig. 6).

**Fig. 6:** Calibration curve of vanillin in the determination of saponin content of samples

**2.7. Data Analysis**

All data sets were analysed using the statistical software package IBM SPSS Statistics 25 for Windows. The dependent variables were subjected to univariate analysis of variance (ANOVA, *P*<0.05) to test the effect of treatments as categorical predictors. Significantly different means were separated by the Posthoc Tukey Multiple Range test (*P*<0.05).

**3. Results**

**3.1. Rhizospheric soil physicochemical properties**

Medium-degree invasion of *C. odorata* led to a significant increase (*P<*0.05) in soil P, while low and high-degree invasion did not show a significant impact on soil P (Table 1). Soil K concentration was significantly higher under low and medium-degree invasion compared to high-degree invasion. Soil Ca concentration significantly increased *(P*<0.05) with a high degree of invasion, whereas Fe concentration significantly decreased (Table 1). Soil Mg concentration significantly increased with the degree of invasion, but there was no significant effect on soil Mn concentration under low, medium, and high degree invasion of *C. odorata*.

Table 1: Physicochemical properties (mineral salts) of soil under different degrees of *C. odorata* invasion (mg/kg)

| **Invasion Degree** | **Phosphorus** | **Potassium** | **Calcium** | **Magnesium** | **Iron** | **Manganese** |
| --- | --- | --- | --- | --- | --- | --- |
| **Uninvaded** | 66.57 ± 7.64**b** | 2834.20 ± 135.49**b** | 286.50 ± 69.96**a** | 53.58 ± 22.45**c** | 3728.20 ± 648.59**a** | 0.23 ± 0.03**a** |
| **Low degree** | 66.38 ± 10.22**b** | 3627.79 ± 153.33**a** | 222.00 ± 23.58**b** | 125.34 ± 12.95**a** | 2626.15 ± 524.05**b** | 0.24 ± 0.01**a** |
| **Medium degree** | 107.47 ± 14.88**a** | 3412.12 ± 362.23**a** | 224.57 ± 17.84**b** | 94.01 ± 33.88**b** | 3020.20 ± 137.37**b** | 0.25 ± 0.02a |
| **High degree** | 71.76 ± 12.14**b** | 2722.03 ± 137.88**b** | 254.95 ± 12.11**ab** | 100.07 ± 12.03**ab** | 1671.41 ± 112.77**c** | 0.24 ± 0.01**a** |

The values in the table are means and standard deviations of the four areas with the same degree of *C. odorata* invasion. Values with different superscript letters in a column indicate significant differences (*P*<0.05). Units are given in mg/kg of sample.

The results obtained showed that soil organic carbon, nitrogen content and total organic matter had significant increases (*P*<0.05) with an increase in the degree of *C. odorata* invasion (Fig. 7), while the invasion situation had no significant effects on soil moisture and pH values.

**Fig. 7:** Effects of different degrees of *C. odorata* invasion on rhizospheric soil organic matter concentrations, pH, and moisture content. Mean values with different superscripts indicate significant differences (*P*<0.05).

**3.2. Bacteria and fungi load**

Bacteria colonies were present in rhizospheric soils of all the different invasion situations of *C. odorata*, with counts ranging from 460000CFU/g at uninvaded soil samples to 670000CFU/g in high degree invaded soils. The invasion of *C. odorata* however did not have a significant difference on the bacterial count of the rhizospheric soil samples at different invasion degrees (Fig. 8a). Fungi count ranged from 1000CFU/g in uninvaded soils to 30000CFU/g in high-degree invaded soils. The total fungi count varied with the degree of invasion, and a high degree of invasion significantly increased the fungal load in the rhizospheric soil (*P*<0.05) (Fig. 8b).

(b)

**Fig. 8**: Effects of different situations of *C. odorata* invasion on bacterial load (a) and total fungi count (b) in rhizospheric soil samples. Mean values with different superscripts indicate significant differences (*P<*0.05).

Bacterial shapes were mainly round, filamentous, and irregular. The form was circular, some with rhizoids and some irregular with entire, undulate, or lobate margins. The colour of the colonies was cream in uninvaded soil samples and white in all degrees of invasion. All samples tested positive for the catalase test and gram test. (Table 2).

Table 2: Different bacterial colonies, their phenotypic and biochemical characterisations, shapes under the microscope and types present in rhizospheric soils at different degrees of C. odorata invasion

| **Invasion situation** | **No of colonies** | **Shapes under the microscope** | **Biochemical characterization** | | **Types of Bacteria** |
| --- | --- | --- | --- | --- | --- |
|  |  |  | Catalase test | Gram test |  |
| **Uninvaded** | 2 | Cocci in clusters and chains  Streptobacillus in chains | Positive | Purple | *Staphylococcus*  *Corynebacterium* |
| **Low degree** | 3 | Cocci in chains and clusters  Streptobacilli in pairs and chains  Cocci bacilli in pairs and clusters | Positive | Purple | *Staphylococcus*  *Corynebacterium*  *Cocci bacillus* |
| **Medium degree** | 1 | Spore forming rods | Positive | Purple | *Bacillus* |
| **High degree** | 2 | Spore-forming rods in chains  Cocci bacilli in clusters, pairs and single | Positive | Purple | *Bacillus*  *Cocci bacillus* |

These various bacterial shapes were not strongly distributed based on invasion degree, meanwhile, the main types observed under the microscope were *Staphylococcus, Corynebacterium, Bacillus* and *Cocci bacillus* (Fig. 10). Based on phenotypic characteristics, samples from all degrees of invasion had growth of fungi that spread, with very raised elevation, white hyphae, black spores, filamentous forms and translucent opacity. Under the microscope, all cultures had sexual and asexual spores, non-septate hyphae, cell walls, rhizoids, and mycelia and were mould-type (Fig. 10h). All degrees of invasion had the growth of *Rhizopus nigricans* fungi.

**Fig. 10.** Microscopic images of the different forms of bacteria observed in rhizospheric soil samples from 1) Uninvaded (a = *Cocci* in clusters and in chains, b*=Streptobacillus* in chains and in clusters., 2) low-degree invasion (c = *Cocci* in chains and clusters*,* d = *Streptobacilli* in pairs and chains*,* e = *Cocci bacillus in pairs and clusters*, 3) medium-degree invasion (f = Spore forming rods) and 4) high-degree invasion of *C. odorata* (g = *Cocci bacillus* in pairs*,* singles and chains and (h) Fungi (Mould fungus) in the Mount Cameroon Region.

**3.3. Mycorrhizal colonization**

The root colonizationof *C. odorata* by mycorrhiza ranged from 23.33 to 50.00, with the highest Arbuscular mycorrhiza fungi (AMF) colonization recorded in root samples from low-degree invasion and the lowest in samples from high-degree invasion. Root colonizationby AMF varied significantly (*P<*0.05), with the degree of invasion (F=5.79), with low-degree invasion resulting in a significant increase in root colonization(Fig. 11).

**Fig. 11:** Effect of different degrees of invasion on the arbuscular mycorrhiza root colonization(%) of *C. odorata*. Data (Mean ± SD) with different letters are significantly different (Tukey’s HSD, *P*<0.05).

**3.4. Phosphatase Activities**

Acid phosphatase activity in the rhizosphere soil of *C. odorata* was found to vary significantly (*P<*0.05), with the degree of invasion (F = 65.94), ranging from 0.69 to 0.90 mmol h^-1^ kg^-1^ (Fig. 12). High-degree invasion led to a significant decrease in phosphatase enzyme activity, while medium-degree invasion resulted in a significant increase (Fig. 12). Moreover, higher acid phosphatase activity was observed in the rhizospheric soil of uninvaded situations compared to low and high-degree invasion situations of *C. odorata*.

**Fig. 12:** Effect of different degrees of invasion on the acid phosphatase activity (mmol/h/kg) in the rhizosphere of *C. odorata*. Data (Mean ± SD) with different letters are significantly different (Tukey’s HSD, *P*<0.05).

**3.5. Correlation between AMF root colonization and acid phosphatase activity of *C. odorata* at different degrees of invasion**

There was a positive correlation (*r*=0.69, *P*<0.05) between *Arbuscula*r mycorrhiza root colonizationand acid phosphatase activity in the rhizospheric soil of *C. odorata.* The rhizospheric soils with increased mycorrhiza root colonizationat varying degrees of invasion had higher acid phosphatase activity (Fig. 13).

**Fig. 13:** Correlation between acid phosphatase activities in the rhizosphere of *C. odorata* and arbuscular mycorrhizal root colonizationat different degrees of invasion

**3.6. Secondary Metabolites**

Secondary metabolites such as flavonoids, saponins, tannins, and phenolic compounds were present in the rhizosphere soil of *C. odorata* at different degrees of invasion, while alkaloids were absent. Low degree invasion significantly increased the concentrations of saponins, flavonoids, and tannins in the soil samples, while higher degrees of invasion significantly reduced their concentrations. The concentration of total phenolic compounds was significantly low in soil samples from low degree invasion. Still, there was a significant increase (*P<*0.05), in its concentration in samples from moderate degree invasion (Fig. 14).

**Fig. 10:** Concentrations of secondary metabolites in rhizospheric soil samples at different degrees of *C. odorata* invasion. Mean values with different letters indicate significant differences (*P*<0.05).

**4. Discussion**

**4.1. Response of rhizosphere soil physicochemical properties to *C odorata* invasion gradients**

Invasive plants can modify soil physicochemical properties and microbial communities to establish themselves in new habitats [30, 40]. The degree of *C. odorata* invasion had significant effects on soil physicochemical properties, which are essential indicators of soil quality for agricultural production and ecosystem maintenance. At a medium degree of invasion, soil P significantly increased, possibly due to high root colonizationby AMF at a low degree of invasion. Studies have shown that rhizosphere microorganisms and mycorrhizal fungi increase P uptake by solubilising or mineralising more P than required, stimulating root growth, or excreting phytase, which breaks down phytate [41]. However, excess soil P can lower P use efficiency and cause environmental risks such as eutrophication of aquatic systems [42].

At a high degree of *C. odorata* invasion, there was a significant drop in soil P, K, and Fe, accompanied by an increase in the microbial load of the rhizospheric soil samples. High microbial load can immobilize P, inhibit root growth, and reduce plant P availability, leading to a drop in soil P content [3, 21]. Phosphorus is essential for photosynthesis, respiration, energy storage and transfer, cell division, and seed formation, and promotes early root formation and growth, improving crop quality [43]. Therefore, reduced soil P content caused by *C. odorata* invasion at low-degree invasion may increase its invasiveness in the area. A drop in the concentration of K in rhizospheric soils can have significant implications for plant growth and water use efficiency. These effects may be minimal within a small range but become more pronounced after several decades of *C. odorata* invasion in the MCR. K plays a crucial role in plant growth as an enzyme activator and helps regulate the plant's water use by controlling stomatal opening and closing [13, 22]. It also maintains the balance of electrical charges at the site of ATP production, promotes the translocation of sugars for plant growth or storage in fruits or roots, is involved in protein synthesis, improves disease resistance, size of grains and seeds, and quality of fruits and vegetables [1].

On the other hand, a drop in Fe concentration in rhizospheric soils can affect the activities of key enzymes such as catalase, peroxidase, cytochrome oxidase, and the synthesis and maintenance of chlorophyll. Under persistent Fe deficiency stress, microorganisms release organic acid anions or siderophores that chelate Fe^3+^ [44]. In contrast, a high degree of *C. odorata* invasion can significantly increase the concentration of soil Ca, which improves carbohydrate transfer and combines with anions, including organic acids, sulphates, and phosphates. This increase can also cause an elevation in soil C, N and organic matter content, which could be attributed to an increase in soil fungi load and a high bacteria count under a high invasion degree. Soil bacteria rectify atmospheric nitrogen, increasing the accessible nitrogen pool available to plants in the rhizosphere [12].

Interestingly, *C. odorata* invasion did not significantly alter soil pH and moisture content at different degrees of invasion, contrary to some previous findings on other invasive plants. Alterations in soil pH and moisture facilitate the invasiveness of some species but were detrimental to the activities of microorganisms [45-46]. The mechanisms by which *C. odorata* invades may thus be different from alteration of soil pH and moisture, requiring further investigations. Presumably the effects of *C. odorata* invasion on soil chemical properties can have significant implications for plant growth, water use efficiency, and soil quality. It is crucial to continue researching the mechanisms by which invasive plants modify soils and the implications for ecosystem maintenance, agricultural production, and human well-being.

**4.2. Dynamics of phosphatase enzyme activity**

At a medium degree of invasion, there was a significant increase in acid phosphatase activities, complemented by an increase in soil P content and AMF colonisation, which could be attributed to improved P cycling [47]. Turner and Haygarth [48], reported that other soil properties such as pH, total N, organic P, and clay content, microbial count, and the amount of organic material present play crucial roles in phosphorus cycling. The contribution of phosphatase-producing *Pseudomonas sp.* is also noteworthy in stimulating enzyme synthesis by intact roots and releasing inorganic phosphate from organic moiety and complex inorganic materials, thereby playing an essential role in the phosphorus cycle [16]. These bacterial species also play essential roles in phosphorus cycling by solubilizing organic and inorganic phosphates into available forms that support plant growth [17].

According to Wang et al. [13], phosphatase enzymes contribute to the cleavage of organic phosphorus, hence supplies organic phosphorus to soil solution by removing a phosphate group from a protein. Interestingly, acid phosphatase activity was high in all degrees of invasion, suggesting its potential as a fallow crop and for the modification of soil biochemical processes. This finding indicates that *C. odorata* invasion can have some benefits in terms of soil fertility management, which could be explored further. Overall, understanding the mechanisms and implications of *C. odorata* invasion on soil processes and plant growth is crucial for addressing the challenges of invasive species management and promoting sustainable agricultural practices.

**4.3. Dynamics of bacterial and fungal load in the rhizosphere**

At a high degree of invasion, it was observed that *C. odorata* invasion did not significantly alter bacterial load but increased fungi load. This finding is intriguing, as the abundance and diversity of microorganisms in the soil play essential roles in maintaining soil fertility [49]. Moreover, microorganisms are recognised as potential indicators of some ecological stresses such as heat, moisture, salts, heavy metals, pesticides, and pollution [50]. Bacterial count was high in all samples. The presence of gram-positive bacteria that tested positive for catalase activities indicates that a majority of the bacteria were strict aerobes or facultative anaerobes such as *Sataphylococcus aureus.* Reports by Sizar et al. [51], revealed that *Staphylococcus* causes food poisoning, toxic shock syndrome, septic arthritis, skin infections, scalded skin syndrome, pneumonia, abscesses, osteomyelitis, inflammatory disease, exfoliative toxin and endocarditis. High amounts of this bacterium in rhizospheric soils of *C. odorata* pose serious human health concerns.

It is, therefore, reasonable to presume that at low degrees of invasion, *C. odorata* might induce environmental stresses that could cause a significant drop in soil fungi load. This observation is consistent with previous studies that have reported altered microbial communities and ecological processes under invasive plant species [52, 1]. The disrupted microbial communities and ecological processes could negatively impact soil fertility, plant growth, and ecosystem functioning. Understanding the mechanisms and implications of *C. odorata* invasion on soil microbial communities could aid in developing effective strategies to manage invasive species and promote sustainable agricultural practices. It could also inform the conservation of biodiversity and ecosystem services.

**4.4. *C. odorata* invasion enhanced Mycorrhizal colonization**

It was observed that at a low degree of invasion, mycorrhizal colonizationwas significantly high, and this correlated positively with enzyme phosphatase activities. This finding is noteworthy, given that several studies have indicated that soil microorganisms and mycorrhizal associations play crucial roles in improving soil fertility through their activities, which oxidise organic matter and promote biogeochemical cycles of C, N, P, and S [12, 53]. The symbiotic relationship between plants and mycorrhizal fungi has been reported to enhance nutrient uptake and stimulate plant growth, particularly in nutrient-poor soils [8-9]. This suggests that the observed increase in mycorrhizal colonizationand phosphatase activities could be attributed to the establishment of mycorrhizal fungi associations in response to the low degree of invasion. The mechanisms and implications of mycorrhizal associations and other soil microbial communities on soil fertility management and plant growth is crucial for addressing the challenges of sustainable agriculture and invasive species management [35, 40]. It could also have significant implications for the conservation of biodiversity and ecosystem services.

**4.5. *C. odorata*** **invasion gradients impacted the concentrations of secondary metabolites**

Observations from the study indicated that low-degree invasion of *C. odorata* significantly increased the concentrations of saponins, flavonoids, and tannins in the soil samples. This may be attributed to the release of root exudates by *C. odorata* during the early stages of the invasion process. Root exudates have been shown to impact soil chemistry, microbial populations, and soil processes and defend the rhizosphere and root against pathogenic microorganisms [54]. The significant increase in the soil concentration of saponins at low degree invasion suggests that *C. odorata* has important potential for leaching remediation, microbial remediation, and phytoremediation, particularly with regards to heavy metal pollutants such as Cd, Zn, Cu, and Pb. Studies have established that high soil concentrations of saponins can remove hydrophobic organic compounds and heavy metals from contaminated soils and water [55,56].

Additionally, the significantly high concentration of flavonoids observed at low degree invasion suggests that *C. odorata* root exudates have antioxidant, anti-inflammatory, anti-cancer, anti-obesity, cardio-protective, and neuroprotective activities. Flavonoids are known to protect plants from biotic and abiotic stresses, act as UV filters, provide drought resistance, and aid in plant acclimation [57-58]. The increased level of tannins observed at low degree invasion is most probably linked to allelopathic responses and poor soil quality. At high degree of invasion, there was a significant drop in the concentration of total phenolic compounds from the rhizospheric soils, which most probably increased soil nitrogen and organic matter by reducing the complexation of proteins. This finding is consistent with previous studies that have reported elevated levels of soil nitrogen and organic matter in soils supplied with phenolic compounds, thus providing an insight into the repeated use of *C. odorata* as a fallow crop in several agroecological ecosystems [59-60].

**5. Conclusion**

The gradient of *C. odorata* invasion had varying effects on soil phosphorus, carbon and nitrogen in rhizospheric soils. It also altered soil fungal load and mycorrhizal colonization together with acid phosphatase enzyme activity and secondary metabolites exudates. *Chromolaena odorata* invades uninvaded areas in mechanisms different from alteration of soil pH and moisture, but an interplay of changes in soil nutrients, microorganisms and release of secondary metabolites. This has varying effects on soil characteristics, with implications for soil quality and ecosystem conservation. This study has demonstrated the significant effects of *C. odorata* invasion on soil ochemical properties, microbial communities, phosphatase activities and secondary metabolites at different gradients of the invasion process. The study highlights the importance of understanding the mechanisms by which invasive species modify soil properties and the impacts on microbial communities and plant growth to inform sustainable land management practices. The results emphasise the interdependence of different components of soil microbiota and the need for further research to understand the complex interactions between invasive species and soil microbiology. Low-degree invasion resulted in increased secretion of flavonoids, saponins and tannins, which provided insights into invasive species' potential benefits and drawbacks on the ecosystem and underscores the importance of continuing research in this field. Further research is needed to improve our understanding of the interplay between different degrees of invasion and soichemical properties, the changes in microbial communities, and the quantification of secondary metabolites for possible medicinal and agricultural purposes.

**Author Contributions**

**Conceptualisation:** Victor NJ, Ndam LM, Fonge BA

**Data curation:** Victor NJ, Tatah BN

**Formal analysis:** Victor NJ, Ndam LM, Tatah BN, Fonge BA

**Methodology:** Victor NJ, Ndam LM, Tatah BN, Fonge BA

**Writing – original draft:** Victor NJ, Tatah BN

**Writing – review & editing:** Victor NJ, Ndam LM, Tatah BN, Fonge BA

**Funding**

This research did not obtain any specific funding or grants from any funding agency.

**Conflict of interest**

The authors declare that they have no financial or personal relationship conflict of interest that relates to the findings reported in this work.

**Acknowledgements**

The authors are grateful to the management bodies of the Agroecology Laboratory, University of Buea, Regional Biocontrol and Applied Microbiology Laboratory of IRAD, Yaoundé and Soil Microbiology Laboratory of the Biotechnology Centre of the University of Yaounde I Cameroon.

**References**

1. Ehrenfeld JG. Ecosystem consequences of biological invasions. Annual Review of *Ecology, Evolution, and* Systematics. 2010; 41(1): 59–80. <http://dx.doi.org/10.1146/annurev-ecolsys-102209-144650>.
2. Stewart BS, Russell AH, Philip AS, Mark JW, Wayne D. Impacts of invasive plants on animal behaviour. *Ecology letters.* 2021; 24(4): 891-907. <http://dx.doi.org/10.1111/ele.13687>.
3. Wekhanya MNN. The effect of invasive species *Lantana camara* on soil nutrients at Ol-Donyo Sabuk National Park, Kenya. Doctoral dissertation, Kenyatta University. 2016. <http://repository.must.ac.ke/handle/123456789/449>.
4. Pisula NL, Meiners SJ. Relative allelopathic potential of invasive plant species in a young disturbed woodland. J. Torrey Bot. Soc. 2010; 137(1): 81-87. <http://dx.doi.org/10.3159/09-RA-040.1>.
5. Perrings C, Dehnen-Schmutz K, Touza J, Williamson M. How to manage biological invasions under globalisation, Trends in Ecology and Evolution. 2005; 20(5): 212-215. <https://doi.org/10.1016/j.tree.2005.02.011>.
6. Waller LP, Allen WJ, Barratt BIP, Condron LM, França FM., Hunt JE et al. Biotic interactions drive ecosystem responses to exotic plant invaders. Science 2020; 368(6494): 967-972. <http://dx.doi.org/10.1126/science.aba2225>.
7. Reinhart KO, Callaway RM. Soil biota and invasive plants. New Phytol. 2006; 170(3):445-457. <https://doi.org/10.1111/j.1469-8137.2006.01715.x>.
8. Hawkes CV, Belnap J, D’Antonio C, Firestone MK. Arbuscular mycorrhizal assemblages in native plant roots change in the presence of exotic grasses Plant Soil. 2006; 281:369-380. <https://doi.org/10.1007/s11104-005-4826-3>.
9. Batten KM, Kate MS, Erin KE. Soil Microbial Community Associated with an Invasive Grass Differentially Impacts Native Plant Performance. Microb Ecol. 2007; 55(2):220-8. <http://dx.doi.org/10.1007/s00248-007-9269-3>.
10. Liu SC, Wang C, Wang L, Dai Z, et al. Different Degrees of Plant Invasion Significantly Affect the Richness of the Soil Fungal Community. PLoS ONE. 2013; 8(12): e85490. <https://doi.org/10.1371/journal.pone.0085490>.
11. Bongard C. Fungal colonizationof the invasive vine *Vincetoxicum rossicum* and native plants. Plant Ecology, Evolution. 2013; 146(1):45-52. <https://doi.org/10.5091/PLECEVO.2013.739>.
12. Sylvia DM, Fuhrmann JJ, Hartel PG, Zuberer DA. Principles and Applications of Soil Microbiology (Pearson Prentice Hall, Upper Saddle River, New Jersey, 2005.
13. Wang Y, Whalen J.K, Chen X, Cao Y, Huang B, Lu C, Shi Y. Mechanisms for altering phosphorus sorption characteristics induced by low molecular weight organic acids. Can. S. Soil Sci. 2016; 96(3):289-298. <http://dx.doi.org/10.1139/CJSS-2015-0068>.
14. George TS, Gregory PJ, Wood M, Read D, Buresh RJ. Phosphatase activity and organic acids in the rhizosphere of potential agroforestry species and maize. Soil Biol. Biochem. 2002; 34(10):1487-1494. <http://dx.doi.org/10.1016/S0038-0717(02)00093-7>.
15. Bhaduri D, Sharmistha P, Purakayastha TJ, Chakraborty K, Yadav RS, Mohd SA. Soil Quality and Plant-Microbe Interactions in the Rhizosphere. © Springer International Publishing Switzerland 2015 E. Lichtfouse (ed.), Sustainable Agriculture Reviews, Sustainable Agriculture Reviews. 2015; 17, <http://dx.doi.org/10.1007/978-3-319-16742-8_9>.
16. Rasol N, Reshi ZA. Effect of the fungicidal Moncozed at different application rates on enzyme activities in a silt loam soil of the Kashmir Himalaya, India. Tropical Ecol. 2010; 51(2): 199-205.
17. Wyszkowska J, Wyszkowski M. Activity of soil dehydrogenase, urease, and acid and alkaline phosphatases in soil polluted with petroleum. J. Toxicol. Environ. Health. A. 2010; 73(17-18): 1202-1210. <http://dx.doi.org/10.1080/15287394.2010.492004>.
18. Ambika SR. Allelopathic plants. 5. *Chromolaena odorata* (L.) King and Robinson. Allelopathy J. 2002; 9(1):35-41.
19. Bani G. Status and management of *Chromolaena odorata* in congo. Proceedings of the fifth international workshop on biological control and management of *Chromolaena odorata,* ARC-PPRI. 2002; 71-73.
20. Sankaran KV, Ellison CA, Suresh TA. Major invasive alien weeds in India: biology and control. 1. Weeds–India–Control, Kerala Forest Research Institute, 632-5, 2009.
21. Vaisakh MN, Pandey A. The Invasive Weed with Healing Properties: A Review on *Chromolaena Odorata*. International Journal of Scientific Studies and Research. 2012; 3(1): 80-83. [www.ijpsr.com](http://www.ijpsr.com/).
22. Wan F, Liu W, Guo J, Qiang S, Li B, Wang J, Yang G et al. Invasive mechanism and control strategy of *Ageratina adenophora* (Sprengel). Science China Life Sciences. 2010; 53(11): 1291-1298. <http://dx.doi.org/10.1007/s11427-010-4080-7>.
23. Kone´ AW, Ettien FE, Jean TG, Aure´lie NA, Laurenza FE et al. Can the shrub *Chromolaena odorata* (Asteraceae) be considered as improving soil biology and plant nutrient availability? Agroforest Syst. 2012;85(2):233–245. <http://dx.doi.org/10.1007/s10457-012-9497-5>.
24. SPREP. Guidelines for invasive species management in the Pacific: a Pacific strategy for managing pests, weeds and other invasive species / compiled by Alan Tye. - Apia, Samoa 20 p.: ill; 29 cm ISBN: 978-982-04-0388-8. 2009.
25. Kus VJ. Invasive Alien Species and their Management Ljubljana, a training course manual, EuroNatur Foundation and Institute Symbiosis. 2021. Available at: <https://savaparks.eu/sava-ties-7448>.
26. Chaudhury J, Mandal UK, Sharma KL, Ghosh H, Mandal B. Assessing soil quality under long-term rice-based cropping system. Commun Soil Sci Plant Anal. 2005; 36(9):1141–1161. <http://dx.doi.org/10.1081/CSS-200056885>.
27. Kah EF, Tchindjang M, Tonye E, Talla T. Combined Supervised Classification methods in Remote Sensing for the Evaluation of forest Dynamics along the slopes of Mount Cameroon. The International Archives of Photogrammetry, Remote Sensing and Spatial Information Science 37 Part B 8. 2008.
28. Fraiser PL, Hall JB, Healey JR. Climate of the Mount Cameroon Region, Long and Medium-term rainfall, temperature and sunshine data. SAFS, University of Bongor MCP-LBG, Limbe1- 56pp. 1998.
29. WWF (World Wide Fund for Nature). Central African 2010 fact sheet, Mount Cameroon National Park, panda.Org. In Muambo and Pedro. An application of geographic information systems to model the habitat suitability of *Pantroglodyte sellioti* in Mount Cameroon National Park in the context of a changing global climate. 2010; 108 (1):63-79.
30. Si C, Liu X, Wang C, Wang L, Dai Z, et al. (2013) Different Degrees of Plant Invasion Significantly Affect the Richness of the Soil Fungal Community. PL0S ONE 2013; 8(12): e85490. <https://doi.org/10.1371/journal.pone.0085490>.
31. Black CA. Method of soil analysis. Amer. Soc. Agronomy, Agronomy No 9 part 2, Madison, Wisconsin, 1965.
32. Jackson ML. Soil chemical analysis Prentice Hall, New York, 1962.
33. Vaze ND, Park S, Brooks AD, Fridman A, Joshi SG. Involvement of multiple stressors induced by non-terminal plasma-charged aerosols during inactivation of air-borne bacteria. PloS ONE. 2017; 12(2): e017/1434. <https://doi.org/10.1371/journal.pone.0171434>.
34. Hanson B, Zhou Y. Bautista EJ, Urch B, Spek M, Silverman F, Muilenberg M, Phipatanakul W, WeinstockG, Sodergren E, Gold DR, Sordillo JE (2016). Characterisation of the bacterial and fungal microbiome in indoor dust and outdoor air samples a pilot study. Environ Sci Process Impacts. 2016; 18(6): 713-24. <https://doi.org/10.1039/c5em00639b>.
35. Vierhelig H, Coughlan A, Wyss U, Piche Y. Ink and vinegar, a simple staining technique for arbuscular mycorrhizal fungi. Appl Environ. Microbiol. 1998; 64(12):5004-5007. <https://doi.org/10.1128%2Faem.64.12.5004-5007.1998>.
36. Van DC, Van der Putten WH, Peters BAM. Plant specific soil-borne diseases contribute to succession in foredune vegetation Nature. 1993; 362(6415):53-56. <https://doi.org/10.1038/362053a0>.
37. Bradford MM. A rapid and sensitive method for quantification of microorganism quantities of proteins utilizing the principle of protein-dye binding. Analytical Biochemistry. 1976; 72 (1-2):248-254. <https://doi.org/10.1016/0003-2697(76)90527-3>.
38. Chlopicka J, Pawel P, Shela G, Aneta J, Pawel Z. Total Phenolic and Flavonoid content, antioxidant activity and sensory evaluation of Pseudocereal breads. LWT-Food Science and Technology. 2012; 46(2):548-555. <https://doi.org/10.1016/j.lwt.2011.11.009>.
39. Yun-Fang T, Wu H, Zhu L. A two-stage estimation method for Random Coefficient Equation Models with Application to Longitudinal HIV Dynamic Data. Statistica Sinica. 2011; 21(3): 1145-1170. <https://doi.org/10.5705/ss.2009.156>.
40. Zubek S, Marta LM, Janusz B, Anna M, Stefa,nowicz, MN, Pawel K. Invasive plants affect arbuscular mycorrhizal fungi abundance and species richness as well as the perfo.rmance of native plants grown in invaded soils. Biol Fertil Soils. Springer. 2016; 52(6):879-893 <https://link.springer.com/article/10.1007/s00374-016-1127-3>.
41. George TS, Simpson RJ, Gregory PJ, Richardson AE. Differential interaction of *Aspergillus niger* and *Peniophora lycii* phytases with soil particles affects the hydrolysis of inositol phosphates. Soil Biology & Biochemistry. 2007; 39(3):793-803. <http://dx.doi.org/10.1016/j.soilbio.2006.09.029>.
42. Zhang T, Page T, Heathwaite L, Beven K, Oliver D, Haygarth P. Estimating phosphorus delivery with its mitigation measures from soil to stream using fuzzy rules. Soil Use and Management. 2013; 29(s1):187-198. <http://dx.doi.org/10.1111/j.1475-2743.2012.00433.x>.
43. Mullins G. Phosphorus, Agriculture and the Environment. Virginia Cooperative Extension, Virginia Tech, Publication 424-029 [www.ext.vt.edu](http://www.ext.vt.edu/), 2018.
44. Neilands JB. Siderophores of bacteria and fungi. Microbiological Sciences. 1984; 1: 9-14. PMID: 6242877
45. Yelenik SG, Stock WD, Richardson DM. Ecosystem level impacts of invasive Acacia saligna in the South African Fynbos. Restor. Ecol. 2004;12(1):44–51. <https://doi.org/10.1111/j.1061-2971.2004.00289.x>.
46. Kuebbing SE, Classen AT, Simberloff D. Two co-occurring invasive woody shrubs alter soil properties and promote subdominant invasive species. J Appl Ecol. 2014; 51:124-133 <http://dx.doi.org/10.1111/1365-2664.12161>.
47. Makoi J, Ndakidemi P (2008) Selected soil enzymes: examples of their potential roles in the ecosystem. Afr. J. Biotechnol. 7(3):181–191. Doi: 10.5897/AJB07.590.
48. Turner B, Haygarth P. Phosphatase activity in temperate pasture soils: potential regulation of labile organic phosphorous turnover by phosphodiesterase activity. Sci Total Environ. 2005; 344(1-3):27-36. <https://doi.org/10.1016/j.scitotenv.2005.02.003>.
49. Nielsen JS, Joner EJ, Declerck S, Olsson S, Jacobsen I. Phospho-imaging as a tool for visualisation and non-invasive measurement of P transport dynamics in arbuscular mycorrhiza. New Phytol. 2002; 154(2):809-819. <http://dx.doi.org/10.1046/j.1469-8137.2002.00412.x>.
50. Bhaduri D, Sharmistha P, Purakayastha TJ, Chakraborty K et al. Soil Quality and Plant-Microbe Interactions in the Rhizosphere. Sustainable Agriculture Reviews. 2015; 17. <https://doi.org/10.1007/978-3-319-16742-8_9>.
51. Sizar O, Leslie SW, Unakal CG. Gram-Positive Bacteria. 2023 May 30. In: StatPearls [Internet]. Treasure Island (FL): StatPearls Publishing; 2024 Jan. PMID: 29261915.
52. Kourtev PS, Ehrenfeld JG, Haggblom M. Exotic plant species alter the microbial community structure and function in the soil Ecology. 2002; 83:3152-3166. <http://dx.doi.org/10.2307/3071850>.
53. Barbosa MAM, Da Silva L, Viana MDG, Coelho NNK, et al. Root contribution to water relations and shoot in two contrasting *Vigna unguiculata* cultivars subjected to water deficit and inoculation. Romanian Agricultural Research. 2013;30: 155–162. [www.incda-fundulea.ro](http://www.incda-fundulea.ro/).
54. Drishty K. Rhizosphere: Hotspot of Soil- Microbes-Plant Interaction. Just Agriculture. 2022; 2(12): e-ISSN: 2582-8223. <http://dx.doi.org/10.1007/978-981-13-7264-3_2>.
55. Zhifeng L, Zhigang Li, Itua Z, Guangming Z, Yunshan L, Mina C et al. Recent advances in the environmental applications of biosurfactant saponins. Journal of environmental chemical engineering. 2017; 5(6):6030-6038. <http://dx.doi.org/10.1016/j.jece.2017.11.021>.
56. Xiao-Lan Y, Yong H. Tea saponins: effective natural surfactants beneficial for soil remediation from preparation to application. RSC advances. 2018;8(43):24312-24321. <http://dx.doi.org/10.1039/C8RA02859A>.
57. Chaves N, Sosa T, Escudero JC. Plant growth inhibiting flavonoids in exudates of Cistus ladanifer and in associated soil. Journal of Chemical Ecology. 2001; 27(3): 623-631. <https://doi.org/10.1023/a:1010388905923>.
58. Amalesh S, Gouranga D, Sanjoy KD. Roles of flavonoids in plants. Carbon. 2011;100 (6):12-35.
59. Djurdjevic L, Pavle P, Mitrovic M. The effect of phenolic compounds on soil properties. J-Soil phenols ER, 31-62, 2009. <https://hdl.handle.net/21.15107/rcub_ibiss_3744>.
60. Misra D, Writuparma D, Gaurave J, Puja R. Interactions and regulatory functions of phenolics in soil-plant-climate Nexus. Agronomy. 2023; 13:280. <http://dx.doi.org/10.13140/RG.2.2.14693.06881>.
