# Supplementary material for: Rhizospheric soil chemical properties and microbial response to a gradient of Chromolaena odorata(L) invasion in the Mount Cameroon Region
Source: PLoS One. 2024 Nov 27;19(11):e0312199. doi: 10.1371/journal.pone.0312199 (PMC11602113; doi:10.1371/journal.pone.0312199)
Supplement: Attachment — Submitted filename: Response to reviewers.docx [file pone.0312199.s003.docx]

Ndam Lawrence Monah
Department of Agronomic and Applied molecular Sciences
Faculty of Agriculture and Veterinary Medicine
University of Buea
4^th^ September, 2024

The editor-in-chief,
PLOS ONE

Dear Sir,
 **Responses to Reviewers` Concerns**

our manuscript No. [PONE-D-24-20731] - [EMID:b56eabaefbd680fb] entitled " Rhizospheric Soil Physicochemical Properties and Microbial Response to a Gradient of *Chromolaena odorata* (L) Invasion in the Mount Cameroon Region" refers. We thank you for the review of our article and the highlighted points to improve on its quality. The reviewers recommended a number of issues to be addressed for clarity and quality. Accordingly, we have addressed the points raised, revised the manuscript and made some rebuttal and clarifications where necessary. It is our hope that these revisions are satisfactory, and we look forward to your publication of the article.

Meanwhile, the responses to reviewers’ comments are attached here below for your appraisal.

Sincerely,
Ndam Lawrence Monah
Corresponding author

**Responses to Reviewers’ comments**

Journal requirements:

1. When submitting your revision, we need you to address these additional requirements.

Please ensure that your manuscript meets PLOS ONE's style requirements, including those for file naming. The PLOS ONE style templates can be found at 
<https://journals.plos.org/plosone/s/file?id=wjVg/PLOSOne_formatting_sample_main_body.pdf> and 
<https://journals.plos.org/plosone/s/file?id=ba62/PLOSOne_formatting_sample_title_authors_affiliations.pdf>.

**Response:**

**Thank you for this comment. The manuscript has been edited and tailored to meet the PLOS ONE's style requirements.**

2. In your Methods section, please provide additional information regarding the permits you obtained for the work. Please ensure you have included the full name of the authority that approved the field site access and, if no permits were required, a brief statement explaining why.

**Response:**

**Thank you for this observation. We have included a statement in the method section that reads as follows:**

**The study investigated an invasive plant species, regarded as a weed, in publicly accessible areas of Mount Cameroon through non-invasive observations and experiments. As it did not involve environmental manipulation or specimen collection for commercial purposes and aimed to enhance ecological understanding and management, no formal permits were necessary under local regulations.**

3. We suggest you thoroughly copyedit your manuscript for language usage, spelling, and grammar. If you do not know anyone who can help you do this, you may wish to consider employing a professional scientific editing service.

The American Journal Experts (AJE) (<https://www.aje.com/>) is one such service that has extensive experience helping authors meet PLOS guidelines and can provide language editing, translation, manuscript formatting, and figure formatting to ensure your manuscript meets our submission guidelines. Please note that having the manuscript copyedited by AJE or any other editing services does not guarantee selection for peer review or acceptance for publication. Upon resubmission, please provide the following:
 
The name of the colleague or the details of the professional service that edited your manuscript

A copy of your manuscript showing your changes by either highlighting them or using track changes (uploaded as a *supporting information* file)

A clean copy of the edited manuscript (uploaded as the new *manuscript* file).

**Response**

**The manuscript has been read again by all authors and the right language was used, and all spelling and grammatical mistakes corrected. Additionally, Prof. Njock Thomas Eku and Dr Ewane Divine of the University of Buea read and edited the manuscript for anomalies with regards to the right language, and all spelling and grammatical errors. A copy of the manuscript showing track changes of all the corrections has been provided. A copy of the clean manuscript has also been provided.**

4. We note that your Data Availability Statement is currently as follows: [All relevant data are within the manuscript and its Supporting Information files]

Please confirm at this time whether or not your submission contains all raw data required to replicate the results of your study. Authors must share the “minimal data set” for their submission. PLOS defines the minimal data set to consist of the data required to replicate all study findings reported in the article, as well as related metadata and methods (<https://journals.plos.org/plosone/s/data-availability#loc-minimal-data-set-definition>).

For example, authors should submit the following data:

- The values behind the means, standard deviations and other measures reported;
- The values used to build graphs;
- The points extracted from images for analysis.

Authors do not need to submit their entire data set if only a portion of the data was used in the reported study.

If your submission does not contain these data, please either upload them as Supporting Information files or deposit them to a stable, public repository and provide us with the relevant URLs, DOIs, or accession numbers. For a list of recommended repositories, please see <https://journals.plos.org/plosone/s/recommended-repositories>.

If there are ethical or legal restrictions on sharing a de-identified data set, please explain them in detail (e.g., data contain potentially sensitive information, data are owned by a third-party organization, etc.) and who has imposed them (e.g., an ethics committee). Please also provide contact information for a data access committee, ethics committee, or other institutional body to which data requests may be sent. If data are owned by a third party, please indicate how others may request data access.

**Response**

**The data used in this research is part of the data collected by a PhD student (Juru Victor, first author of the MS) pending the defense of his thesis. It is a tradition of the institution (University of Buea) that data be shared upon request, after a student has effectively defended his/her PhD thesis and submitted a copy of the thesis to the Library. Attached is a document from the institution on data sharing.**

5. We note that Figure(s) 2 and 10 in your submission contain copyrighted images. All PLOS content is published under the Creative Commons Attribution License (CC BY 4.0), which means that the manuscript, images, and Supporting Information files will be freely available online, and any third party is permitted to access, download, copy, distribute, and use these materials in any way, even commercially, with proper attribution. For more information, see our copyright guidelines: <http://journals.plos.org/plosone/s/licenses-and-copyright>.
 
We require you to either (1) present written permission from the copyright holder to publish these figures specifically under the CC BY 4.0 license, or (2) remove the figures from your submission:
 
a. You may seek permission from the original copyright holder of Figure(s) 2 and 10  to publish the content specifically under the CC BY 4.0 license. 
 
We recommend that you contact the original copyright holder with the Content Permission Form (<http://journals.plos.org/plosone/s/file?id=7c09/content-permission-form.pdf>) and the following text:
“I request permission for the open-access journal PLOS ONE to publish XXX under the Creative Commons Attribution License (CCAL) CC BY 4.0 (<http://creativecommons.org/licenses/by/4.0/>). Please be aware that this license allows unrestricted use and distribution, even commercially, by third parties. Please reply and provide explicit written permission to publish XXX under a CC BY license and complete the attached form.”
 
Please upload the completed Content Permission Form or other proof of granted permissions as an ""Other"" file with your submission. 
 
In the figure caption of the copyrighted figure, please include the following text: “Reprinted from [ref] under a CC BY license, with permission from [name of publisher], original copyright [original copyright year].”
 
b. If you are unable to obtain permission from the original copyright holder to publish these figures under the CC BY 4.0 license or if the copyright holder’s requirements are incompatible with the CC BY 4.0 license, please either i) remove the figure or ii) supply a replacement figure that complies with the CC BY 4.0 license. Please check copyright information on all replacement figures and update the figure caption with source information. If applicable, please specify in the figure caption text when a figure is similar but not identical to the original image and is therefore for illustrative purposes only.

**Response**

**Thank you for this comment. Figures 2 and 10 have been removed from the manuscript.**

**ADDITIONAL COMMENTS FROM JOURNAL AFTER FIRST RESUBMISSION**

**IMPORTANT: PLEASE DO NOT REPLY TO THIS EMAIL**

**If you are unable to complete any points that are requested in this email, please explain why in the 'Enter Comments' tab of the online submission form prior to re-submitting your manuscript. This will let us quickly assess your response and send your manuscript to an Academic Editor as soon as possible.**

**=============================================**

**PONE-D-24-20731R1**

**Rhizospheric Soil Chemical Properties and Microbial Response to a Gradient of Chromolaena odorata(L) Invasion in the Mount Cameroon Region**

**Dr Lawrence Monah Ndam**

**Dear Dr. Ndam,**

**We've checked your submission and before we can proceed, we need you to address the following issues:**

**1. In the online submission form, you indicated that "The data used in this research is part of the data collected by a PhD student (Juru Victor, first author of the MS) pending the defense of his thesis. It is a tradition of the institution (University of Buea) that data be shared upon request, after a student has effectively defended his/her PhD thesis and submitted a copy of the thesis to the library. Attached is a document from the institution on data sharing."**

**All PLOS journals now require all data underlying the findings described in their manuscript to be freely available to other researchers, either**

**1. In a public repository,**

**2. Within the manuscript itself, or**

**3. Uploaded as supplementary information.**

**This policy applies to all data except where public deposition would breach compliance with the protocol approved by your research ethics board. If your data cannot be made publicly available for ethical or legal reasons (e.g., public availability would compromise patient privacy), please explain your reasons on resubmission and your exemption request will be escalated for approval.**

**Response**

**All relevant data are within the manuscript and its Supporting Information files.**

**2. We note that Figure 1 in your submission contain copyrighted images. All PLOS content is published under the Creative Commons Attribution License (CC BY 4.0), which means that the manuscript, images, and Supporting Information files will be freely available online, and any third party is permitted to access, download, copy, distribute, and use these materials in any way, even commercially, with proper attribution. For more information, see our copyright guidelines: http://journals.plos.org/plosone/s/licenses-and-copyright.**

**Response**

**Thank you for this comment. Figure 1 has been removed from the manuscript.**

[Note: HTML markup is below. Please do not edit.]

Reviewers' comments:

Reviewer's Responses to Questions

**Comments to the Author**

1. Is the manuscript technically sound, and do the data support the conclusions?

The manuscript must describe a technically sound piece of scientific research with data that supports the conclusions. Experiments must have been conducted rigorously, with appropriate controls, replication, and sample sizes. The conclusions must be drawn appropriately based on the data presented.

Reviewer #1: Partly

Reviewer #2: Yes

Reviewer #3: No

2. Has the statistical analysis been performed appropriately and rigorously?

Reviewer #1: I Don't Know

Reviewer #2: Yes

Reviewer #3: Yes

3. Have the authors made all data underlying the findings in their manuscript fully available?

The [PLOS Data policy](http://www.plosone.org/static/policies.action#sharing) requires authors to make all data underlying the findings described in their manuscript fully available without restriction, with rare exception (please refer to the Data Availability Statement in the manuscript PDF file). The data should be provided as part of the manuscript or its supporting information, or deposited to a public repository. For example, in addition to summary statistics, the data points behind means, medians and variance measures should be available. If there are restrictions on publicly sharing data—e.g. participant privacy or use of data from a third party—those must be specified.

Reviewer #1: No

Reviewer #2: Yes

Reviewer #3: Yes

4. Is the manuscript presented in an intelligible fashion and written in standard English?

PLOS ONE does not copyedit accepted manuscripts, so the language in submitted articles must be clear, correct, and unambiguous. Any typographical or grammatical errors should be corrected at revision, so please note any specific errors here.

Reviewer #1: Yes

Reviewer #2: Yes

Reviewer #3: Yes

5. Review Comments to the Author

Please use the space provided to explain your answers to the questions above. You may also include additional comments for the author, including concerns about dual publication, research ethics, or publication ethics. (Please upload your review as an attachment if it exceeds 20,000 characters)

Reviewer #1: The author kept saying physicochemical properties of rhizospheric soil in the manuscript but only chemical properties were determined and of course biochemical and microbiological properties of the rhizosphere soil. For your manuscript to qualify as "physicochemical", physical properties of soil have to be included. Such properties as bulk density, infiltration, particle density, mean weight diameter, porosity, aggregate stability, particle size distributions e.t.c. have to be included. I suggest therefore, the word "Physicochemical" should not be used as suggested in the reviewed manuscript uploaded herewith.

**Response**

**Thank you for this keen observation. The word “Physicochemical” properties have been rephrased to “Chemical” Properties through out the manuscript as requested.**

Reviewer #2: Review comments: PONE-D-24-20731
This study determined the effects of gradient densities of plant invasion on the soil properties, enzyme activities and population differences of microbial communities in field. The study showed many data of soil properties, enzyme activities, secondary metabolites and the bacterial and fungal population densities. These data analyzed in this manuscript are interesting. However, there were several shortcomings of the manuscript written in this style. I suggest this manuscript a major revision before publication.

General comments:

Introduction: paragraph 4 and 5 (line 71-88) could be rewritten before paragraph 2 (……Invasive species release root exudates……).

**Response**

**Thank you for this key comment to make the introduction better. In the introduction, paragraph 4 and 5 (line 71-88) have been rewritten before paragraph 2 as requested.**

Methods
Line 136 to 138, soil samples collected with 5 cm of C. odorata rhizosphere from the four study areas. I think these samples are bulk soils but not rhizosphere soils (mm). Please check and improve.

**Response**

**Thank you for your observation. This has been revised as suggested for easy understanding by the readers and the word “rhizosphere” has been changed to “around the root zone of C. odorata” as that is the exact position where the soil was collected.**

Line 256, The phenolic components and flavonoids from soils collected were determined. Therefore, these could not be referred as root exudates in this study. Different invasion density of this plant could affect the composition contents of soil secondary metabolites. But whether these secondary metabolites were secreted by C. odorata were not determined.

**Response**

**This is a key observation that has been applied in the appropriate sections of the manuscript to indicate that the variation in the concentration of the allelochemicals is not as a result of the root exudates but it is as a result of the different invasion densities of C. odorata in different localities.**

Results

The photos were poorly generated by Excel. Please improve.

**Response**

**Thank you for this comment. All figures generated in excel have been ameliorated as requested for proper visibility and clarity**

The results of contents of secondary metabolites affected by the gradient of plant invasion density were interesting. Correlation between them could be analyzed and improved in the discussion section.

**Response**

**The correlation between these secondary metabolites has been generated in table 3 and the discussion section improved base on these correlations as requested.**

Specific comments:
The calibration curves of secondary metabolites (Fig.3 to 6) could be listed in the supplied materials.
Some words were misspelled such as pH, nitrogen, organic matter, in Fig.7. Please check and improve.

Response

**Thank you for your thoughtful comments regarding our manuscript.**

1. **Calibration Curves: We appreciate your suggestion to include the calibration curves of secondary metabolites (Figures 3 to 6) in the supplementary materials. However, we believe it is essential to retain these curves in the Methods section. The calibration curves play a critical role in the analysis of the various secondary metabolites, providing necessary context for understanding the methodology employed in our study. By keeping them in the main text, we ensure that readers can easily refer to this important information while reviewing our results.**
2. **Spelling Corrections: We acknowledge your observation regarding the misspelled terms in Figure 7, specifically "pH," "nitrogen," and "organic matter." We have carefully reviewed the figure and make the corrections to ensure accurate spelling and clarity.**

Reviewer #3: Comments for Author,

Thank you for your submission. Even the paper is interessting, there are some issue to improve this paper.

Please obtained results are unclear. Please add your future recommandation end of conclusion remark.

Discussion is superficial. Please reconsider all obtained results and support with data analyses.

**Response**

**Thank you for your valuable feedback on our manuscript. We are grateful for your recognition of the intriguing aspects of our work and for your recommendations for improvement.**

**Clarity of Results: We understand the necessity of clearly presenting our results. In response, we have revised the results section to improve clarity and ensure that our findings are effectively communicated. Furthermore, we have added recommendations for future research at the conclusion to offer a forward-looking perspective on our work.**

**Depth of Discussion: We appreciate your insights regarding the discussion's superficiality. We have taken your comments to heart and have upgraded this section. All results have been reconsidered, and we have incorporated more detailed data analyses to back up our interpretations. This improvement aims to provide a more thorough exploration of our findings and their implications.**

Best regards

6. PLOS authors have the option to publish the peer review history of their article ([what does this mean?](https://journals.plos.org/plosone/s/editorial-and-peer-review-process#loc-peer-review-history)). If published, this will include your full peer review and any attached files.


If you choose “no”, your identity will remain anonymous but your review may still be made public.


**Do you want your identity to be public for this peer review?** For information about this choice, including consent withdrawal, please see our [Privacy Policy](https://www.plos.org/privacy-policy).

Reviewer #1: **Yes:** Ibrahim A Aliyu (PhD)

Reviewer #2: No

Reviewer #3: No

[NOTE: If reviewer comments were submitted as an attachment file, they will be attached to this email and accessible via the submission site. Please log into your account, locate the manuscript record, and check for the action link "View Attachments". If this link does not appear, there are no attachment files.]

While revising your submission, please upload your figure files to the Preflight Analysis and Conversion Engine (PACE) digital diagnostic tool, <https://pacev2.apexcovantage.com/>. PACE helps ensure that figures meet PLOS requirements. To use PACE, you must first register as a user. Registration is free. Then, login and navigate to the UPLOAD tab, where you will find detailed instructions on how to use the tool. If you encounter any issues or have any questions when using PACE, please email PLOS at [figures@plos.org](mailto:figures@plos.org). Please note that Supporting Information files do not need this step.
